# IL-33 and soluble ST2 in follicular fluid are associated with premature ovarian insufficiency

**DOI:** 10.3389/fendo.2024.1463371

**Published:** 2024-12-06

**Authors:** Maoxing Tang, Xuedong Sun, Ping Li, Weifen Deng, Xi Zhan, Peng Sun, Yuhua Shi

**Affiliations:** ^1^ Department of Reproductive Medicine, Guangdong Provincial People’s Hospital (Guangdong Academy of Medical Sciences), Southern Medical University, Guangzhou, China; ^2^ Department of Neurology, Nanfang Hospital, Southern Medical University, Guangzhou, China; ^3^ Department of Reproductive Medicine, Women and Children’s Hospital, School of Medicine, Xiamen University, Xiamen, China; ^4^ Reproductive Medicine Centre, Shenzhen Hengsheng Hospital, Shenzhen, China; ^5^ Department of Obstetrics and Gynecology, Center for Reproductive Medicine, Nanfang Hospital, Southern Medical University, Guangzhou, China

**Keywords:** premature ovarian insufficiency, IL-33, soluble ST2, inflammation, predictor, ovarian reserve, embryo development, embryo quality

## Abstract

**Background:**

Premature ovarian insufficiency (POI) is a common reproductive disease that is associated with chronic inflammation in ovaries. Interleukin 33 (IL-33) is a pro-inflammatory IL-1 family cytokine, and functions as an alarmin reflecting inflammatory reaction. Our study aimed to investigate levels of IL-33 and its soluble receptor (sST2) in both follicular fluid (FF) and paired serum during different stages of POI, and evaluate their predictive potentials for POI. Furthermore, we attempted to determine whether IL-33 and sST2 were associated with embryo quality.

**Methods:**

A total of 148 women, including 50 patients with biochemical POI (bPOI) (10 IU/L < follicle-stimulating hormone (FSH) ≤ 25 IU/L), 46 patients with POI (25 IU/L<FSH ≤ 40 IU/L) and 52 age-matched control women with normal ovarian reserve were involved in this study. FF and paired serum were collected from these women. IL-33 and sST2 were measured using quantitative sandwich enzyme-linked immunosorbent assay.

**Results:**

FF IL-33 levels were significantly increased in bPOI and POI patients compared to controls. They exhibited positive associations with FSH and luteinizing hormone (LH), whereas negative correlations with anti-Müllerian hormone (AMH), estradiol (E_2_), testosterone (T) and antral follicle count (AFC). Receiver operating characteristic (ROC) curve analysis showed that for POI prediction, FF IL-33 had a better predictive accuracy (AUC 0.901) with high sensitivity (82.61%) and good specificity (84.62%) than those for bPOI prediction. IL-33 levels in paired serum did not differ among three groups. Regarding sST2, its levels in FF declined with POI progression. Contrarily, they showed negative associations with FSH and LH, but positive correlations with AMH, E2, T and AFC. ROC analysis revealed that FF sST2 had comparatively weak potentials for both bPOI and POI prediction compared to those of FF IL-33. Similarly, there was no significant alteration of sST2 in paired serum among three groups. Additionally, Spearman’s correlation analysis revealed that FF IL-33 levels were negatively associated with the rates of Day-3 good-quality embryos (r=-0.206, *P*=0.012), whereas FF sST2 did not.

**Conclusion:**

Our study revealed an increased abundance of FF IL-33, whereas an sST2 deficiency with POI development. This implies that IL-33 and sST2 levels might be associated with the development of POI.

## 1 Introduction

Premature ovarian insufficiency (POI) is a common reproductive endocrine disorder affecting 1-5% of reproductive-aged women (1). POI is characterized by the early onset of ovarian dysfunction in women younger than the age of 40 years. It is often manifested by menstrual disorders like amenorrhea or oligomenorrhea, and hormone level disruption with increased follicle-stimulating hormone (FSH) whereas decreased estradiol (E_2_) (1). Clinically, POI is regarded as a progressive state of ovarian insufficiency (2). It usually involves three consecutive stages from occult, and biochemical or precursor to overt ovarian insufficiency (2, 3). Ultimately, POI can lead to ovarian failure, female infertility, osteoporosis, cardiovascular disease and even neurodegenerative disorder (1). The known causes of POI involve a variety of aspects, such as genetic, metabolic, autoimmune, environmental pollution and iatrogenic factors. And yet a proportion of cases are idiopathic in origin (1). To date, there is, however, no therapy available to cure the POI patients (4). Moreover, the markers for diagnosis and prediction of POI, particularly for cases at early stage of POI, remain limited (3, 4). As such, the development of novel markers aimed at predicting and monitoring ovarian function in POI remains an alluring prospect.

While the pathogenesis of POI is not fully elucidated, it has been reported to be associated with a sterile chronic inflammation within the ovary (5–9). Along with POI arising from certain etiologies, like autoimmune, genetic, metabolic, and iatrogenic factors, both human and animal studies reveal that reactive oxygen species (ROS) accumulates, leading to excessive oxidative stress in ovaries (10–12). Subsequently, ROS triggers nucleotide-binding domain and leucine rich repeat containing family, pyrin domain containing 3 (NLRP3) inflammasome activation, which boosts an excessive production of multiple pro-inflammatory cytokines, including IL-1β and IL-18 (13–15). Recent studies using mouse POI models indicate that over the course of POI, the levels of pro-inflammatory cytokines, involving IL-1α/β, IL-6 and TNF-α, were remarkably increased in both serum and ovary (16). In turn, these cytokines adversely affect follicle reserve and ovarian function (9, 16, 17). Consequently, the intensity and magnitude of ovarian inflammatory responses and their associations to the patient’s manifestation of POI seem to be relevant issues (2, 18, 19).

Interleukin 33 (IL-33) is a recently identified pro-inflammatory protein that belongs to the IL-1 family of cytokines. It has a molecular weight of 30 kDa (20). Its unique receptor is the suppressor of tumorgenicity 2 (ST2), also known as IL-33R and IL1RL1 (21). It exists in two forms: a transmembrane form known as ST2L, and a soluble form known as sST2. As a pleiotropic cytokine, IL-33 binds the ST2L that can subsequently induce either type 1 or type 2 helper T cell (Th) inflammation (21–24). It has been shown that both IL-33 and ST2 are constitutively expressed in both immune and non-immune cells within diverse tissues (22, 25). In response to tissue/cell damage, infection, necrosis, or oxidative stress, IL-33 is released by damaged or necrotic cells, which functions to regulate physiological and pathological processes related to chronic inflammation (20, 24, 26). Intriguingly, upon tissue-derived stimuli such as inflammatory factors, sST2 can be released from various cell types (25). It acts as a decoy receptor to inhibit the IL-33 signaling pathway (22, 25). Mounting evidence has revealed that IL-33 levels are elevated within the serum or tissue under inflammation-associated diseased conditions, such as heart failure, atherosclerosis, asthma, sepsis, trauma and pulmonary fibrosis (20, 25, 27). Similar changes are also observed in sST2 under these inflammatory conditions (25, 28). Thus, IL-33 seems to be an alarmin cytokine with essential roles in promoting chronic inflammation (22, 24, 26).

At the female reproductive system of mouse and human, IL-33 and ST2 are found to be expressed in uterus, endometrium as well as ovary (22, 29). So far, their roles in both reproductive physiologies and pathologies are not fully understood. Several studies have revealed that IL-33 and ST2 are associated with endometriosis, which is known as a chronic inflammatory gynecological disorder (30, 31). Their results showed that IL-33 levels were increased in both the plasma and peritoneal fluid of patients with severe endometriosis compared with healthy controls (30, 31). For sST2, an elevation was also found in peritoneal fluid of endometriosis patients in comparison to healthy controls (31). By contrast, although available data in the literature concerning the roles of IL-33/ST2 in POI is lacking, accumulated evidence has indicated vital roles of IL-33/ST2 in multiple aspects of ovarian physiology, including folliculogenesis, ovulation and follicular atresia (29, 32, 33). Given that IL-33 is closely associated with inflammation-associated diseases, its dynamic variation during the progression of ovarian insufficiency might mirror ovarian reserve and function for POI patients.

Therefore, in the current study, we aimed to investigate the levels of IL-33 and sST2 in both follicular fluid and paired serum during different stages of POI. Next, we explored whether their levels are correlated with POI progression. Finally, we attempted to determine whether IL-33 and sST2 levels are associated with embryo quality.

## 2 Materials and methods

### 2.1 Participants

This study recruited a total of 148 women at the Reproductive center of Guangdong Provincial People’s Hospital of Southern Medical University from December 2022 to June 2024. They were further divided into three groups, including biochemical POI (bPOI) (n=50), POI (n=46) and controls (n=52) based on the following criteria. The study was approved by the Ethics Committee of Guangdong Provincial People’s Hospital of Southern Medical University (NO. KY-Z-2022-379-01). Written informed consent was obtained from all subjects before entering the study.

The inclusion criteria for bPOI known as a precursor stage of POI involved: (i) younger than 40 years old, (ii) regular or irregular menstruation, and (iii) two elevated basal FSH levels (10 IU/L < FSH ≤ 25 IU/L) with an interval beyond 4 weeks (3). For POI, the patients met the criteria including oligomenorrhea or amenorrhea for at least 4 months, as well as an increased FSH level >25 IU/L found on two occasions beyond 4 weeks apart. The controls included women with regular menses and normal ovarian reserve who experienced assisted reproductive treatment owing to tubal or male factor. Meanwhile, they were matched with bPOI and POI patients for age and body mass index (BMI). Additionally, women with other endocrine or autoimmune disorders (e.g. polycystic ovary syndrome, hyperprolactinemia, thyroiditis and systemic lupus erythematosus), history of ovarian surgery or chemoradiotherapy, chromosomal abnormality and severe systemic diseases were excluded. The clinical characteristics of the three groups were collected (**Table 1**).

**Table 1:** Baseline characteristics of the participants.

Characteristics	Control (n=52)	bPOI (n=50)	POI (n=46)	*P* value
Age (y)	31.62 ± 2.76	31.50 ± 3.09	31.87 ± 1.49	0.8046
BMI (kg/m^2^)	21.66 ± 2.13	21.12 ± 2.04	21.76 ± 2.32	0.3807
FSH (IU/L)	5.88 ± 1.015	15.51 ± 1.67	34.30 ± 11.71	<0.001
LH (IU/L)	4.03 ± 0.87	5.17 ± 1.63	19.22 ± 10.39	<0.001
E_2_ (pg/mL)	36.73 ± 7.61	36.35 ± 9.53	15.65 ± 5.77	<0.001
T (ng/mL)	0.24 ± 0.04	0.20 ± 0.05	0.16 ± 0.04	<0.001
AMH (ng/mL)	3.13 ± 0.77	0.97 ± 0.75	0.33 ± 0.37	<0.001
AFC	12.96 ± 1.66	4.14 ± 1.31	1.52 ± 0.98	<0.001

Data are presented as mean and standard deviation of the mean (S.D.).

### 2.2 Collection of serum and follicular fluid

Peripheral blood samples were obtained into individual sterile vacutainer during days 2-4 of the menstrual cycle or collected randomly for patients with amenorrhea. The samples were kept undisturbed at room temperature for at least 30 minutes, followed by a centrifugation at 3000 rpm for 10 minutes at 4°C. Then, they were stored at -80°C until the assay. Paired follicular fluid samples were obtained from dominant follicles having oocytes with a diameter at least 14 mm during oocyte retrieval, based upon the standard procedures as previously described and stored at -80°C until detection (3).

### 2.3 Endocrine hormone measurement and transvaginal ultrasonography

The levels of endocrine hormones of all subjects, including anti-Müllerian hormone (AMH), FSH, luteinizing hormone (LH), estradiol (E2), and total testosterone (T) were measured using chemiluminescence immunoassay (Beckman) during days 2-4 of the menstrual cycle. Antral follicle count (AFC) was determined by transvaginal ultrasonography during days 2-4 of the menstrual cycle. The AFC referred to the number of bilateral ovarian follicles with a diameter of 2-10 mm at the early stage of follicular development.

### 2.4 Assessment of human embryo quality

At the day 3 following fertilization, the embryo quality was evaluated based upon the Society for Assisted Reproductive Technology (SART) grading system (34). The embryos possessing ≥6 blastomeres with a fragmentation degree <25% were considered as Day-3 good-quality embryos (34). The rate of Day-3 good-quality embryo was referred to the number of Day-3 good-quality embryos divided by the number of normal zygotes with two pronucleus.

### 2.5 Quantitative determination of IL-33 and sST2 concentrations by enzyme-linked immunosorbent assay (ELISA)

The concentrations of IL-33 in serum and follicular fluid (FF) were analyzed using a human IL-33 Quantikine^®^ ELISA kit (R&D Systems, Inc., Minneapolis, MN, USA) according to the manufacturer’s instructions. The detectable limit of the kit is 0.357 pg/mL. The concentrations of sST2 in serum and FF were measured using a human ST2/IL-33 R Quantikine^®^ ELISA kit (R&D Systems, Inc., Minneapolis, MN, USA) based on the manufacturer’s instructions. The detection limit of the kit is 5.1 pg/mL. For each assay, both standards and samples were run in duplicate.

### 2.6 Statistical analysis

All statistical analyses were performed using the GraphPad Prism software (v9.00, GraphPad, San Diego, CA, USA) and SPSS (version 23.0; SPSS Inc.). The normality of data distribution was assessed by Kolmogorov-Smirnov test. The continuous variables conforming to a normal distribution were presented as mean and standard deviation of the mean (S.D.), and were analyzed using Student’s *t*-test or one-way analysis of variance. The Mann-Whitney *U*-test was applied for the variables not normally distributed. In addition, the association between different variables with ovarian reserve markers was estimated by Pearson correlation analysis. The Spearman’s correlation test was used to evaluate the correlation between the variables with the rates of Day-3 good-quality embryos. The receiver operating characteristic (ROC) curve was performed to assess the predictive potential of different markers. *P*<0.05 was considered statistically significant.

## 3 Results

### 3.1 Baseline and reproductive characteristics

A total of 148 women including 52 control women, 50 patients with bPOI, and 46 patients with POI were enrolled for this study. The baseline and reproductive characteristics of all the participants are presented in **Table 1**. There were no statistically significant differences in age and BMI at recruitment among the three groups (*P*>0.05). As expected, the levels of FSH and LH were remarkably increased, whereas the levels of E_2_, T, AMH and bilateral AFC were significantly decreased along with the progression of ovarian insufficiency (all *P*<0.001).

### 3.2 Levels of IL-33 in follicular fluid and paired serum of women at different stages of ovarian insufficiency

**Figure 1:**
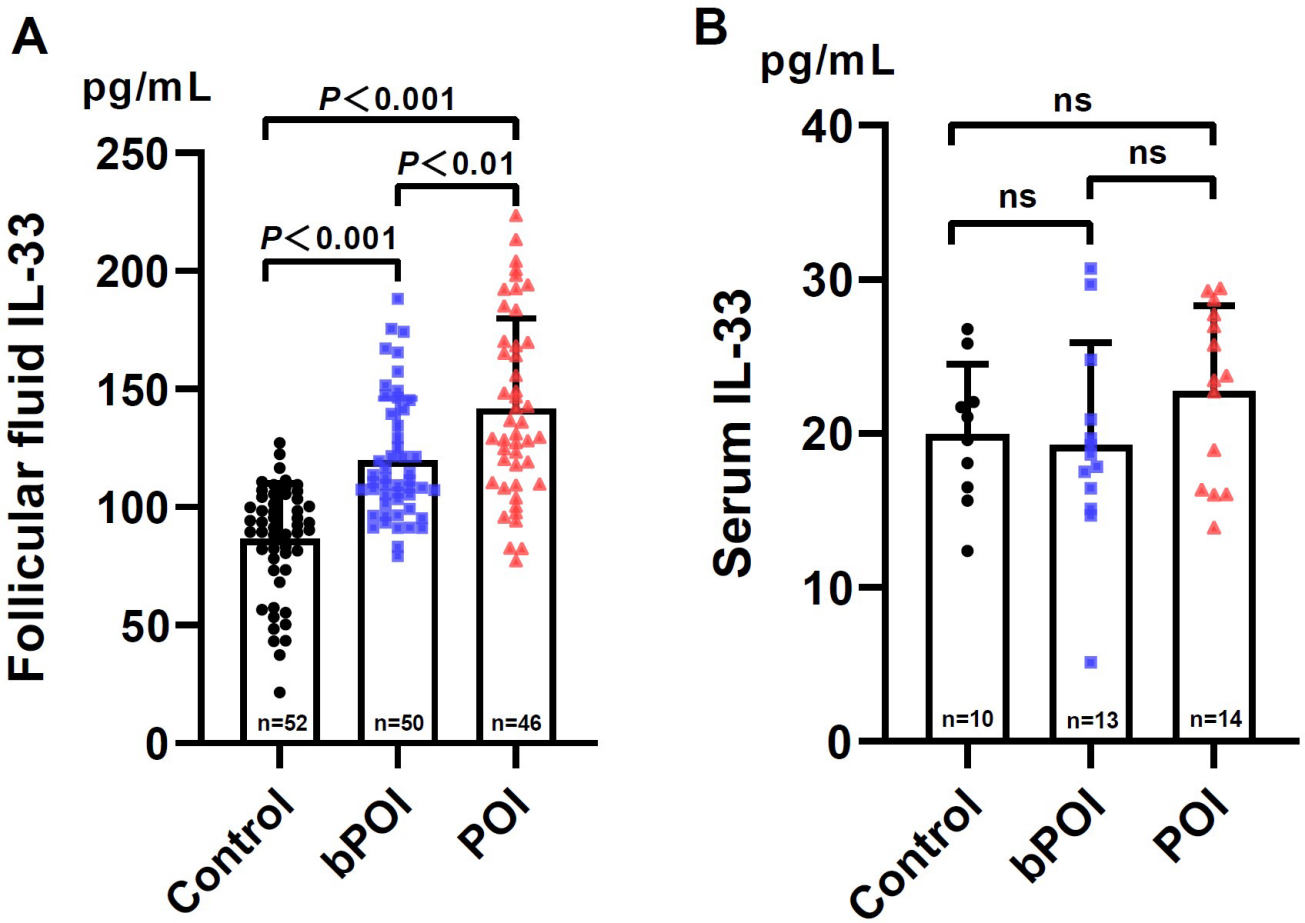
Levels of IL-33 in follicular fluid **(A)** and paired serum **(B)** in controls and women at different stages of ovarian insufficiency (bPOI, biochemical premature ovarian insufficiency; POI, premature ovarian insufficiency). Data are given as mean ± SD; n shows the total number of samples tested from each group. Differences between means were determined by the Mann-Whitney test. *P*<0.05 was considered significant; ns, not statistically significant.

Initially, we measured the levels of IL-33 in follicular fluid (FF) collected from women with bPOI and POI, as well as the controls (**Figure 1A**). We found that in comparison to the controls, IL-33 levels were conspicuously elevated in women with bPOI and POI (both *P*<0.001). Additionally, there was a higher level of IL-33 in FF in POI group than those of bPOI group (*P*<0.01). Subsequently, we further examined the IL-33 levels in the paired serum of patients with bPOI and POI, and the controls. It’s noteworthy that as many as 111 (out of 148) subjects had IL-33 levels below the detection limit of the ELISA kit. Therefore, only 37 subjects with detectable levels of IL-33 were included for data analysis. The serum levels of IL-33 were significantly lower than those in FF. There was no statistically significant difference in serum IL-33 levels among the three groups (*P*>0.05) (**Figure 1B**).

**Figure 2:**
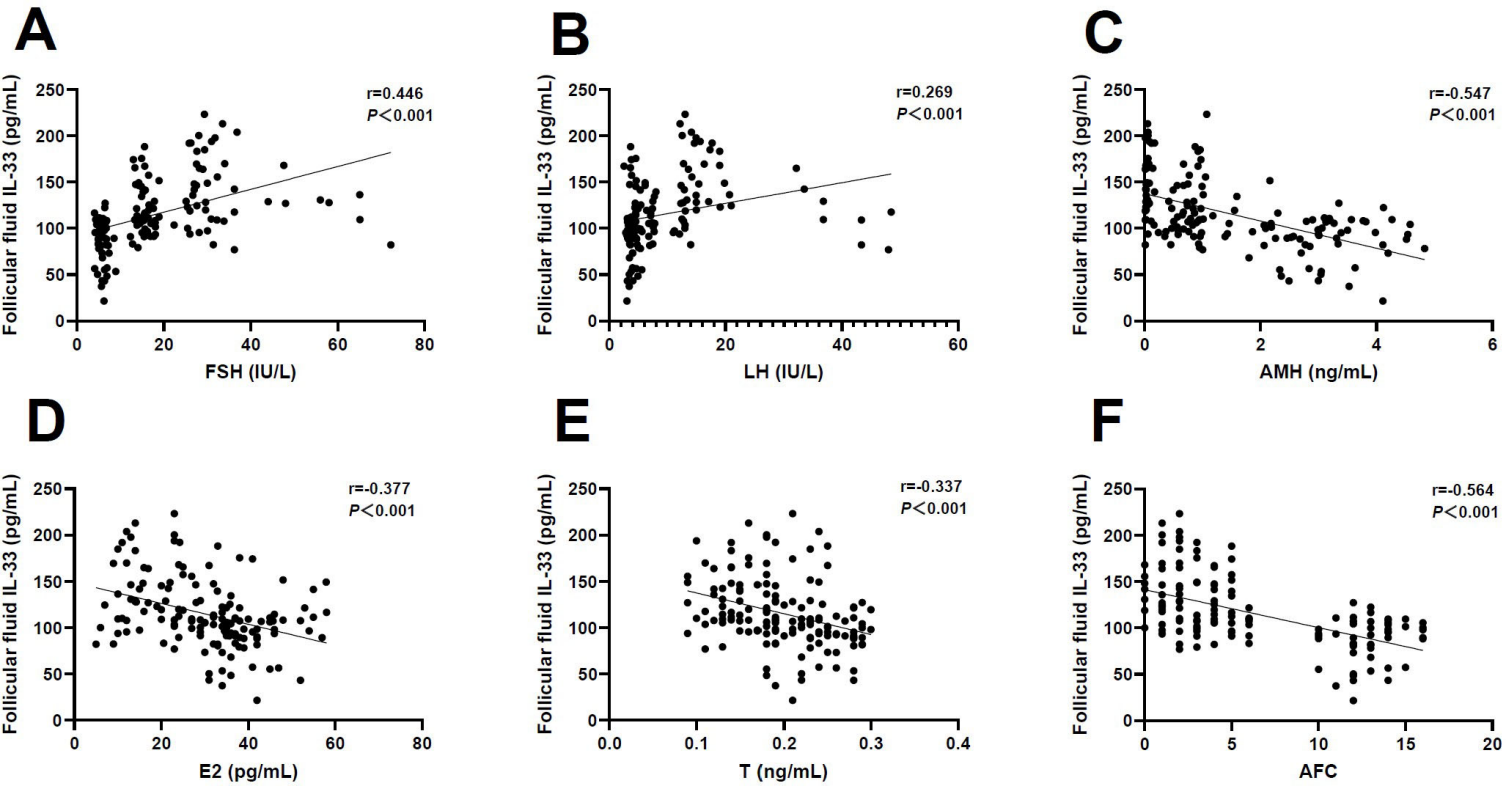
Correlation analysis of follicular fluid IL-33 and ovarian reserve markers. **(A)** Follicle-stimulating hormone (FSH) (r=0.446, *P*<0.001). **(B)** Luteinizing hormone (LH) (r=0.269, *P*<0.001). **(C)** Anti-Müllerian hormone (AMH) (r=-0.547, *P*<0.001). **(D)** Estradiol (E2) (r=-0.377, *P*<0.001). **(E)** Testosterone (T) (r=-0.337, *P*<0.001). **(F)** Antral follicle count (AFC) (r=-0.564, *P*<0.001).

Next, we determine the correlation between FF IL-33 levels and ovarian reserve markers. Interestingly, we observed that the FF level of IL-33 was positively associated with FSH (r=0.446, *P*<0.001) and LH (r=0.269, *P*<0.001) (**Figures 2A, B**), but negatively associated with AMH (r=-0.547, *P*<0.001), E_2_ (r=-0.377, *P*<0.001), T (r=-0.337, *P*<0.001), and AFC (r=-0.564, *P*<0.001), respectively (**Figures 2C–F**). Together, these results indicate an association between FF IL-33 levels and ovarian reserve markers commonly applied in POI.

### 3.3 Predictive potential of follicular fluid IL-33 for bPOI and POI

Given the difference in FF IL-33 among the three groups, we further examined the predictive potential of FF IL-33 for bPOI and POI using ROC curve analysis (**Table 2**). We found that for prediction of bPOI, the area under curve (AUC) of FF IL-33 was 0.828 with relatively high sensitivity (84.00%), but with unsatisfactory specificity (61.54%). In contrast, FF IL-33 exhibited inferior performance compared to AMH (AUC 0.965). Furthermore, for POI prediction, the FF IL-33 showed a better predictive accuracy (AUC 0.901) with high sensitivity (82.61%) and good specificity (84.62%). However, it was not as good as AMH (AUC 0.982) with higher sensitivity (97.83%) and good specificity (92.31%). These findings suggest that the FF IL-33 may serve as a potential predictor for POI progression.

**Table 2:** ROC curve analysis for bPOI and POI prediction.

Groups	Variables	AUC	Sensitivity	Specificity	*P* value
bPOI	IL-33	0.828	84.00%	61.54%	<0.001
	sST2	0.705	90.00%	44.23%	<0.001
	AMH	0.965	90.00%	96.15%	<0.001
POI	IL-33	0.901	82.61%	84.62%	<0.001
	sST2	0.763	71.74%	73.08%	<0.001
	AMH	0.982	97.83%	92.31%	<0.001

### 3.4 Levels of sST2 in follicular fluid and paired serum of women at different stages of ovarian insufficiency

**Figure 3:**
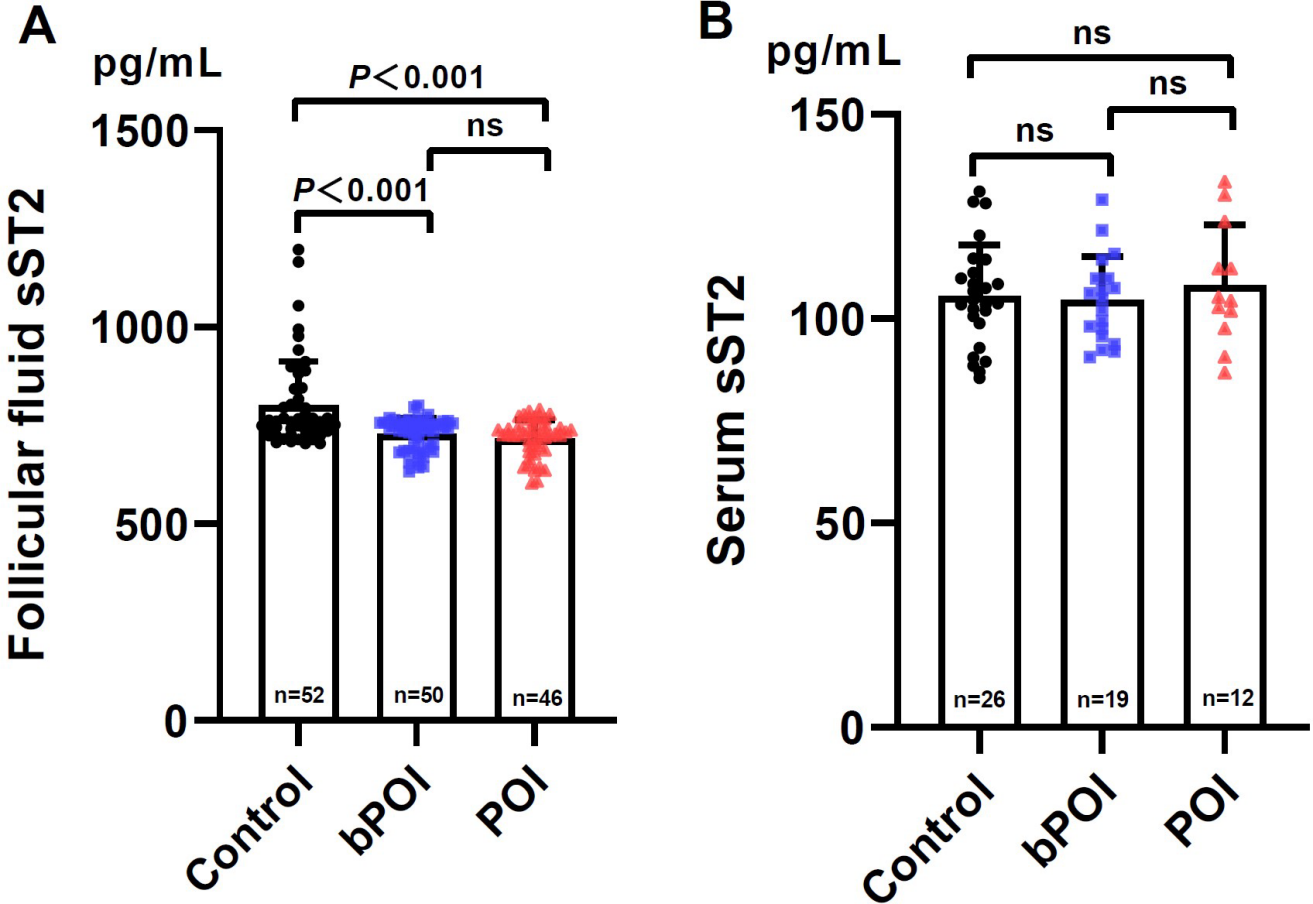
Levels of sST2 in follicular fluid **(A)** and paired serum **(B)** in controls and women at different stages of ovarian insufficiency (bPOI, biochemical premature ovarian insufficiency; POI, premature ovarian insufficiency). Data are given as mean ± SD; n shows the total number of samples tested from each group. Differences between means were determined by the Mann-Whitney test. *P*<0.05 was considered significant; ns, not statistically significant.

In parallel, we firstly tested FF sST2 levels in control women and patients with bPOI and POI. It was revealed that the FF levels of sST2 in bPOI and POI groups were remarkably lower than those of controls (both *P*<0.001) (**Figure 3A**). However, FF sST2 levels did not differ between bPOI and POI groups (*P*>0.05) (**Figure 3A**). Secondly, we also analyzed the sST2 levels in the paired serum collected from the three groups. The data showed that only 57 (out of 148) subjects had detectable serum sST2. The overall levels of serum sST2 were significantly low, an approximately sevenfold decreased over the levels found in FF (**Figure 3B**). No significant difference was observed among the three groups (**Figure 3B**).

**Figure 4:**
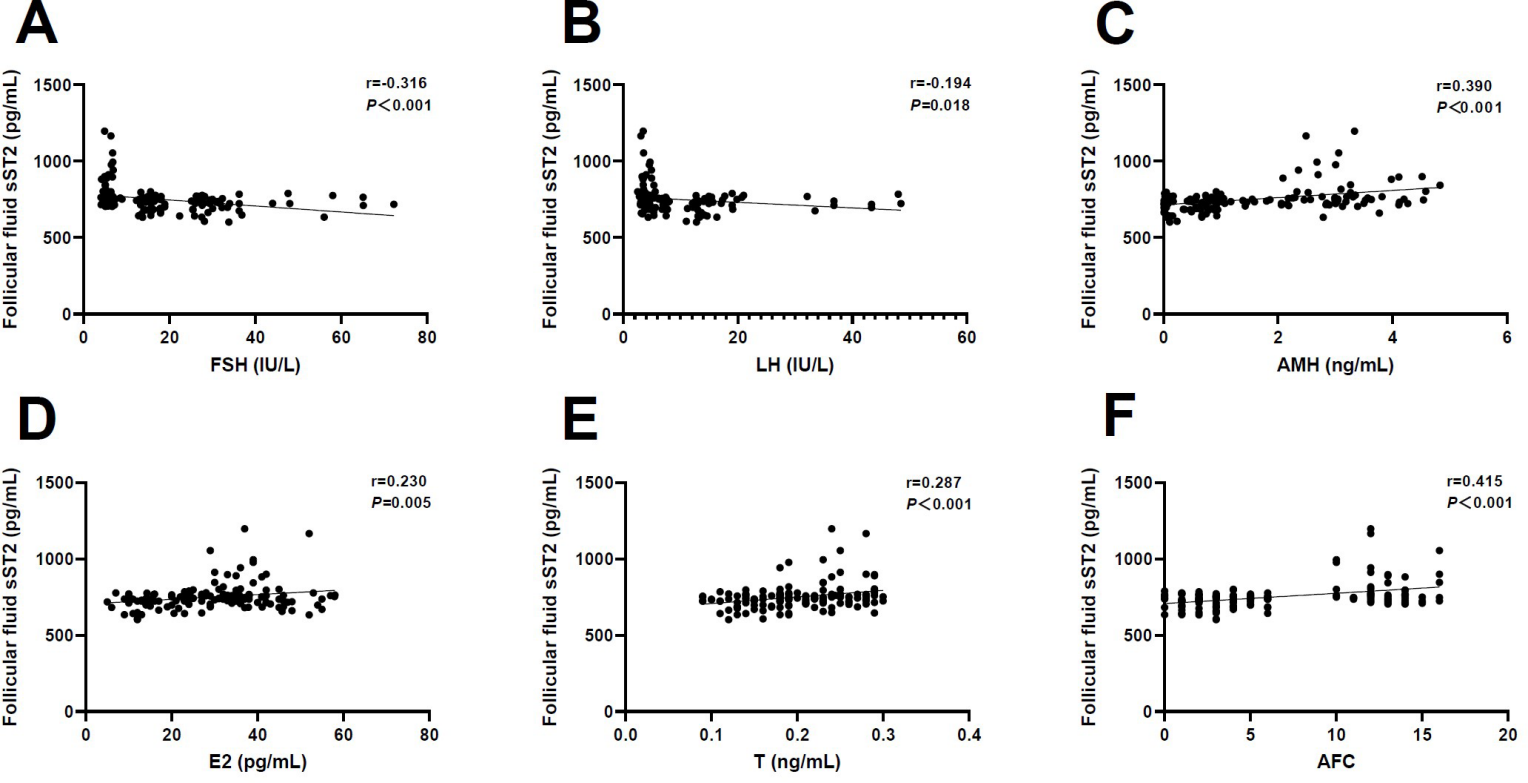
Correlation analysis of follicular fluid sST2 and ovarian reserve markers. **(A)** Follicle-stimulating hormone (FSH) (r=-0.316, *P*<0.001). **(B)** Luteinizing hormone (LH) (r=-0.194, *P*=0.018). **(C)** Anti-Müllerian hormone (AMH) (r=0.390, *P*<0.001). **(D)** Estradiol (E2) (r=0.230, *P*=0.005). **(E)** Testosterone (T) (r=0.287, *P*<0.001). **(F)** Antral follicle count (AFC) (r=0.415, *P*<0.001).

Likewise, we examined whether FF sST2 levels were associated with ovarian reserve markers. It was found that there were negative associations between FF sST2 levels with FSH (r=-0.316, *P*<0.001) and LH (r=-0.194, *P*=0.018) (**Figures 4A, B**). By contrast, the FF levels of sST2 were positively associated with AMH (r=0.390, *P*<0.001), E2 (r=0.230, *P*=0.005), T (r=0.287, *P*<0.001), and AFC (r=0.415, *P*<0.001), respectively (**Figures 4C–F**). These observations indicate that FF sST2 levels were in relevance with ovarian reserve during POI development. However, given the small number of samples examined, our observations require careful interpretation.

### 3.5 Predictive potential of follicular fluid sST2 for bPOI and POI

Upon the alterations of FF sST2 along with POI progression, we further tested whether the levels of FF sST2 had potentials for bPOI and POI prediction by ROC curve analysis (**Table 2**). For prediction of bPOI, the AUC of FF sST2 was 0.705 with satisfactory sensitivity (90.00%), whereas with poor specificity (44.23%). In addition, for POI prediction, the FF sST2 exhibited a declined predictive accuracy (AUC 0.763) with lower sensitivity (71.74%) and specificity (73.08%) than those of FF IL-33 and AMH. Overall, while FF sST2 levels were correlated with POI development, they had comparatively weak potentials for both bPOI and POI prediction.

### 3.6 Association between follicular fluid IL-33 and sST2 with the rates of Day-3 good-quality embryos

As aforementioned, there were associations between FF IL-33 and sST2 with ovarian reserve markers. We wonder whether they can synchronously mirror the quality of oocytes and embryos. Given that the rates of Day-3 good-quality embryos were reportedly to reflect oocyte/embryo quality, and were strong predictors for reproductive outcomes including pregnancy and implantation rates (35), we therefore further explored whether FF IL-33 and sST2 were correlated with rates of Day-3 good-quality embryos. We found that FF IL-33 levels turned out to be negatively associated with the rates of Day-3 good-quality embryos (r=-0.206, *P*=0.012). By contrast, there was no significant association between FF sST2 levels and the rates of Day-3 good-quality embryos (r=0.084, *P*=0.309). Collectively, our data suggest that IL-33 in FF might affect embryo developmental competence.

## 4 Discussion

Due to the premature ovarian functional decay and unclear etiology, women with POI remain a challenging group of infertility patients (4). There are several therapies for POI in the clinical setting, including psychological and family genetic counseling, hormone-replacement therapy (HRT), oocyte donation and cryopreservation (1). However, these methods mainly focus on managing the symptoms of POI patients and have certain limitations. To date, there is no effective treatment for recovering ovarian function in women with POI (1, 4). These indications suggest that timely and accurate diagnosis of POI are crucial, especially for those patients within early stage of ovarian insufficiency. This can ensure adequate time for the clinician to make treatment options for POI patients. Currently, the primary biomarkers to predict and assess ovarian function of POI patients mainly originated from ovarian follicles and granulosa cells, like E_2_, AMH and inhibin B (1, 3). Although chronic inflammatory milieu in ovaries have been reported in women with POI, the inflammatory markers identified for POI indications are still lacking (5, 7, 9, 36).

As a pro-inflammatory IL-1 family cytokine, IL-33 is initially identified as an alarmin or danger signal within a variety of tissues (22, 37). Normally, IL-33 is stored in the nucleus, but is frequently released from both the nucleus and cell in response to tissue damage, necrosis, oxidative stress, inflammation and infection (20, 22). Subsequently, it can stimulate a MyD88- and nuclear factor-kappa B (NF-κB)-dependent inflammatory reaction through its surface receptor called ST2 (20, 38). Aberrant expressions of IL-33 and/or ST2 has been well documented in plentiful inflammatory disorders, such as systemic lupus erythematosus, rheumatoid arthritis, multiple sclerosis, and inflammatory bowel disease (37). However, the roles of IL-33 and sST2 in patients with POI has not been elucidated (22). To our knowledge, the present study for the first time reveals that, IL-33 in follicular fluid, but not in serum, continuously increases, while sST2 has an opposing alteration along with the development of POI. This is further reinforced by their strong associations with other ovarian reserve markers, including FSH, AMH and AFC. In addition, the levels of IL-33 and sST2 in follicular fluid might serve as potential predictors for both bPOI and POI. Hence, our results indicate that follicular fluid IL-33 and sST2, as inflammation-associated factors, may be related with POI progression.

In females, IL-33 and ST2 are found to be present in the uterus, like cervical, endometrial, myometrial tissues of mice and humans (22, 32). Besides, they are constitutively expressed in ovarian tissues, including the ovarian follicles, granulosa cells of primordial, primary, secondary and antral follicles, ovarian stroma as well as corpus lutea (22, 32). Under the physiological condition, both IL-33 and ST2 expressions are shown to be modulated by fluctuations of ovarian hormones (e.g. estrogen and progesterone) along with menstrual cycles or estrous cycles (32). It has been revealed that within the ovary, the IL-33-ST2 signaling pathway plays an important role in folliculogenesis, ovulation and atretic follicle disposal (22, 29, 38). The physiological concentrations of IL-33 and ST2 appear to be required for these ovarian events (29, 32, 38). Notably, mice with IL-33 deficiency by blockage or knock-out of IL-33 exhibit reduced follicular pool, ovulation and phagocytotic activity, whereas accumulated catabolic waste in ovaries (29, 33, 38). Accordingly, the IL-33-ST2 signaling system might act to maintain the ovarian homeostasis and functions.

Given that POI is a continuum of compromised ovarian function, we wonder whether IL-33 is associated with the development of ovarian insufficiency. Thus, in our study, we assessed the levels of IL-33 in both follicular fluid and serum obtained from bPOI, POI patients and healthy controls. The data showed that comparing with controls, the concentration of follicular fluid IL-33 was greatly enriched with the progression of POI. By contrast, its concentration in serum was undetectable in most cases of bPOI, POI and control groups. So far, there is less data regarding IL-33 and POI in the literature, making it difficult to compare our results with others’. Nevertheless, IL-33 has been studied in other reproductive disorders including endometriosis and polycystic ovarian syndrome (PCOS) (30, 39, 40). Both of them are well understood to be characterized by chronic inflammation (40–42). An increased abundance of serum IL-33 was found in PCOS patients in comparison to healthy fertile controls (39). Similarly, several human studies have revealed that IL-33 levels were elevated in plasma, serum, peritoneal fluid and endometriotic tissues of women with endometriosis compared with healthy controls (30, 31, 40, 43). However, under the pathological condition, direct evidence in the literature to unravel the negative effects of IL-33 on ovarian function is currently lacking. Using human stromal cells derived from ovarian endometrioma (hOVEN-SCs), a recent work further demonstrated that IL-33 expression in these cells was upregulated by 17β-estradiol through estrogen receptor pathway. In turn, high levels of IL-33 enhanced invasion ability of hOVEN-SCs via ST2/mitogen-activated protein kinase (MAPK)/matrix metalloproteinase MMP-9 signaling pathway, ultimately contributing to ovarian dysfunctions and endometriosis progression (44). Remarkably, mouse model studies revealed that supplementation of recombinant IL-33 exacerbated ovarian dysfunctions and endometriosis via promoting inflammation and fibrosis within tissues (40, 43). Collectively, our results were consistent with these findings, indicating that IL-33 might drive inflammation in ovarian microenvironment, and its abundance may be in relation to disease activity during POI development. It was revealed that IL-33-ST2 axis can stimulate inflammation response by activating the MAPK and NF-κB pathway (24, 44, 45). Emerging evidence has suggested the roles of IL-33-ST2 axis in several inflammatory diseases like asthma, endometriosis, pulmonary and ovarian fibrosis (9, 44, 46, 47). As abundant IL-33 was present in POI ovaries, we speculate that IL-33 might drive the ovarian inflammation via MAPK signaling pathway during POI progression. This hypothesis is, however, not determined in our work, which represents a limitation of the present study. Therefore, further investigations to determine this issue as well as the source of IL-33 within the milieu of POI ovaries are required. Additionally, future studies will be necessary to delineate the functional role of IL-33 in the POI pathogenesis.

It is well known that sST2, a soluble form of ST, lacks the transmembrane and cytoplasmic regions (31, 48). It acts as a decoy receptor for inhibiting IL-33 activity by blocking IL-33 signaling through ST2L (20, 31). As such, sST2 often directs anti-inflammatory properties within diverse tissues (22, 31). In the present study, we found declined levels of follicular fluid sST2 in both bPOI and POI groups comparing with controls, albeit they remained abundant in all groups. But by comparison, serum sST2 levels were relatively lower, and no significant alteration was found among the three groups. Until now, the role of sST2 in the critical ovarian events like folliculogenesis remains poorly understood (22). A previous study analyzed sST2 expression in the follicular fluid obtained from infertility women who underwent *in vitro* fertilization or intra-cytoplasmic sperm injection (IVF/ICSI) due to female, male, mixed or unexplained factors (49). In agreement with our data, this study revealed that sST2 was more abundant in follicular fluid than those in plasma (approximately 7.9-fold) (49). Notably, higher levels of follicular fluid sST2 were found in larger follicles as well as in retrieval oocytes which developed to good-quality embryos than those which are in small size and graded average embryos, respectively (49). Furthermore, *ex vivo* experiments in this study showed that human granulosa cells secreted sST2. They might be a source of sST2 in ovaries during oocyte/embryo development (49). These findings suggest that physiological sST2 levels in the ovaries are associated with follicular development. Recent mice studies revealed that ovarian sST2 expressions were modulated by reproductive hormones, as reflected by their dynamic changes throughout the estrous cycle (32). Nevertheless, the molecular mechanisms underpinning sST2 modulation in ovarian physiological processes are still unknown. Future investigations are required to determine the precise roles of sST2 in ovaries.

Disturbed levels of sST2 have been reported in the context of reproductive pathologies, like endometriosis, pre-eclampsia (PE), recurrent spontaneous abortion (RSA), and preterm labor (PRL) (22, 50, 51). However, these studies have inconsistent results. Some studies showed an elevated serum level of sST2 in patients with RSA and PE (50, 52). Also, sST2 levels were increased in the peritoneal fluid of patients with endometriosis (31). Contrarily, another study found that women with PRL had diminished sST2 in the amniotic fluid (51). Current understanding of sST2 in reproductive pathologies is still limited, and as yet sST2 has not been investigated in POI (22). Our work is the first to examine the changes of follicular fluid sST2 concentration in POI patients. Unlike IL-33, a decreased level of sST2 in the follicular fluid was observed to be correlated with POI progression. This is in contrast to IL-33 which, as aforementioned, is elevated in the follicular fluid along with POI development. As a soluble receptor of IL-33, sST2 has anti-inflammatory properties through binding to IL-33, and limit the bioactivity of IL-33 (22, 53). Consequently, a possible explanation for our data is that sST2 deficiency in local ovarian environment fails to dampen the pro-inflammatory effects driven by IL-33, ultimately leading to POI occurrence. In line with our findings, a previous study has showed that preterm labor (PRL) patients caused by intra-amniotic infection or inflammation had diminished concentrations of sST2 in amniotic fluid comparing with those who delivered at term (51). This implicates that a decline in sST2 likely augments the inflammatory reaction. Hence, these findings further support our hypothesis that sST2 may function as a counter-regulatory mechanism for balancing IL-33 abundance involved in the POI pathogenesis. The deficiency of sST2 could induce exacerbated the inflammatory response within ovaries and aggravated POI progression. However, available data concerning potential adverse effects of sST2 overexpression on ovarian functions is still lacking. Accordingly, further studies are required to clarify this issue and the regulatory mechanisms responsible for sST2 secretion during POI.

Owing to the irreversible decline of ovarian function in women with POI and no curable approaches for these patients, timely diagnosis and even prediction of POI are of significance, which require multiple reliable markers throughout the spectrum of POI (1, 3, 4). In the clinical practice, the current markers applied for POI patients are mainly focused on ovarian reserve, involving AFC, FSH, E2, AMH and inhibin B (1, 4). Given that IL-33-sST2 system might reflect inflammatory status and involve in essential ovarian events within ovaries with POI development, it is of particular interest to determine whether follicular fluid IL-33 or sST2 could serve as a predictor for POI. Our data showed that comparing with sST2, follicular fluid IL-33 had a better predictive accuracy for bPOI and POI, but inferior to AMH. Furthermore, our results indicated that there were strong associations of follicular fluid IL-33 and sST2 with FSH, E_2_, AMH and AFC. It’s well established that the follicular microenvironment is essential for folliculogenesis and subsequent oocyte development (9, 19). Previous studies have showed that high levels of pro-inflammatory cytokines in follicular fluid, like IL-1, IL-6 and IL-18, could compromise oocyte/embryo development (16, 17, 54). In agreement with these findings, the present study showed that follicular fluid IL-33 levels were negatively associated with the rates of Day-3 good-quality embryos, whereas sST2 did not. This indicates that elevated IL-33 derived local follicular inflammation was relevant with declined quality of oocytes and embryos. Additionally, it’s noteworthy that IL-33 or sST2, as a follicular fluid predictor, differs from the commonly used indicators for ovarian reserve. This is likely because they may specifically mirror the ovarian inflammatory milieu, which affects ovarian function and follicle developmental competence. Although our data are preliminary, they are intriguing, and suggest that IL-33 and sST2 in follicular fluid might become supplementary indicators for an overall evaluation of POI, as well as for a comprehensive assessment of the effectiveness of these patients’ treatment. Nevertheless, our study was limited by the small sample size, future work is still required to examine the potentials of IL-33 or sST2 becoming a biomarker for POI by using randomized clinical trial with a large sample size and suitable statistical models.

## 5 Conclusion

In summary, the present study first revealed an increased abundance of follicular fluid IL-33, whereas an sST2 deficiency in women with POI. This implied that IL-33 and sST2 might be associated with POI development. However, our conclusion was limited due to a small number of human samples analyzed. Future researches on the roles of IL-33-sST2 signaling system in regulating follicular development, steroidogenesis, and POI pathogenesis is warranted.

## Data Availability

The raw data supporting the conclusions of this article will be made available by the authors, without undue reservation.
